# Acute Tubular Necrosis Following Renal Transplantation: A Case Report

**DOI:** 10.7759/cureus.93380

**Published:** 2025-09-27

**Authors:** Md. Rezaul Alam, Ferdous Jahan, A H Hamid Ahmed, Muhammad Nazrul Islam, Rana Mokarram Hossain, Md. Omar Faroque, Syed Fazlul Islam, Md. Kabir Hossain, A. K. M Shahidur Rahman, Md. Masudul Karim, S. M. Remin Rafi, Tashnia Tamanna Chowdhury, Nur-E- Hasna

**Affiliations:** 1 Nephrology, Bangladesh Medical University (BMU), Dhaka, BGD

**Keywords:** acute tubular necrosis (atn), calcineurin inhibitors (cni), cyclosporine-induced nephrotoxicity, delayed graft function (dgf), renal transplantation

## Abstract

Acute tubular necrosis (ATN) remains one of the most frequent causes of delayed graft function (DGF) following renal transplantation, particularly in the early postoperative period. It is commonly associated with ischemia-reperfusion injury, prolonged cold or warm ischemia time, donor-related hemodynamic instability, and the nephrotoxic effects of calcineurin inhibitors (CNI) such as cyclosporine. ATN contributes significantly to early post-transplant morbidity, impacts long-term graft survival, and poses diagnostic challenges due to its clinical overlap with acute rejection. Cyclosporine-induced nephrotoxicity can result in severe tubular injury, leading to ATN, particularly in the early post-transplant period. Despite its efficacy in preventing graft rejection, cyclosporine's toxic effects on the renal vasculature and tubular cells can exacerbate ischemia-reperfusion injury, prolong graft dysfunction, and increase the need for dialysis. Here, we report a case of ATN following renal transplantation in a patient who was on cyclosporine.

## Introduction

Delayed graft function (DGF) commonly occurs following a kidney transplant. It is typically defined as the requirement for dialysis within the first seven days after the transplant. The incidence of DGF is approximately 25% in recipients of kidneys from deceased donors, while it is reported to be as low as 5% in those receiving kidneys from living donors [1,2]. Acute tubular necrosis (ATN) is one of the important reasons for DGF. ATN is a condition that involves a sudden decline in the kidney’s ability to filter waste products due to a drop in the glomerular filtration rate (GFR), leading to the accumulation of waste products in the body [3]. ATN represents the predominant cause of acute renal failure occurring within the initial weeks following renal transplantation [4]. It was reported that the incidence of ATN varied from 0 to 27% in cases of kidney transplantation from a living donor, while the incidence rate in cadaveric donors ranges from 14 to 57% [4]. On average, the incidence of ATN is notably higher among recipients of cadaveric donor kidneys, affecting roughly 34% of cases, compared to an incidence of approximately 9% in recipients of kidneys from living donors [4]. Several factors are responsible for these wide variations, such as warm ischemia time, types of perfusion fluid, perfusion technique (machine or manual perfusion), donor hypotension, reduced allograft blood flow, or use of nephrotoxic medications [5]. Evidence suggests that ATN is the leading cause of graft failure in up to 50% of kidney transplant recipients, particularly when cyclosporine is used [6]. When ATN occurs, patients often need to undergo dialysis, and cyclosporine must be reduced by 50% [7].

## Case presentation

A 44-year-old male with end-stage renal disease (ESRD) due to glomerulonephritis (GN) with hypertension (HTN), got renal allograft transplantation on 17 March 2025. The donor was his younger brother, an apparently healthy man of 35 years old. On pretransplant evaluation, there was one haplotype match, tissue crossmatch was negative, and cytomegalovirus (CMV) immunoglobulin G (IgG) was positive for both donor and recipient. Warm ischemic time was two minutes, and cold ischemic time was 24 minutes. The postoperative period was uneventful. Induction was given by basiliximab on day 0 and day 4, along with initially intravenous (IV) methyl prednisolone for three days, tablet ciclosporine 200 mg 12 hourly (7.5 mg/kg/day), tablet mycophenolate mofetil (MMF) 540 mg twice daily. The postoperative period was uneventful till the 3rd postoperative day (POD) (Table 1).

**Table 1:** Intake-output and renal status at different postoperative days (POD)

POD	Intake (mL)	Output (mL)	Drain (mL)	Creatinine (mg/dL) (Reference range: 0.6–1.3 mg/dL)
Day 0	11,300	14,950	200	2.1
1^st^ POD	8200	8050	175	1.5
2^nd^ POD	8350	7480	50	1.3
3^rd^ POD	6850	6550	30	1.1
4^th^ POD	2575	483	100	1.4
5^th^ POD	1370	62	100	3.8
6^th^ POD	1100	9	70	6.5
10^th^ POD	1150	630	-	6.1
12^th^ POD	1670	960	-	5.2
14^th^ POD	2040	1890	-	4.3
16^th^ POD	2250	3500	-	3
18^th^ POD	8230	8720	-	2.4
20^th^ POD	7550	9310	-	1.3

On the 4th POD morning, the patient suddenly developed severe right calf pain and decreased output (Table 1). Immediately, catheter flushing was done, and a fluid challenge was given. However, no improvement. A Doppler study was done on the same day, which revealed right sapheno-femoral thrombosis.

**Figure 1:**
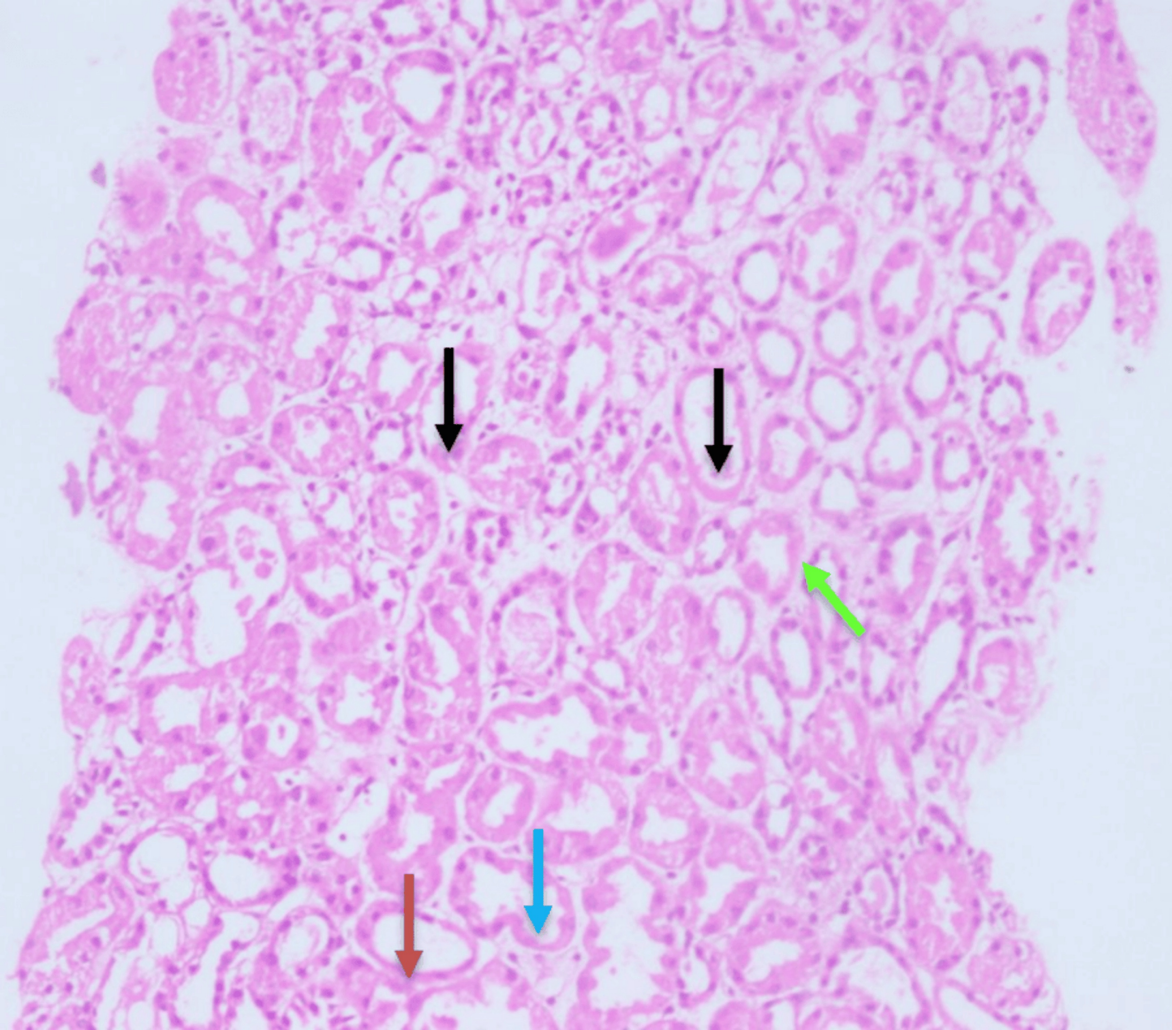
Histopathology of renal tissue, showing loss of tubular epithelial cells with tubular dilation (red arrow), vacuolization of tubular epithelial cells (blue arrows), sloughing of tubular cells into the lumen (black arrows), widely spaced tubules indicating interstitial edema (parrot green arrow) (hematoxylin and eosin, x10)

Urine routine microscopic examination (RME) was sent on the 4th POD, which revealed protein 4+, pus cell 20-25/high power field (HPF), and red blood cell (RBC) - plenty/HPF. Subsequently, we managed the patient with injection methylprednisolone 500 mg for five days (21 March 2025 to 26 March 2025). After getting all reports to confirm sapheno-femoral thrombosis, low molecular weight heparin (LMWH) was initiated immediately on 22 March 2025, which was switched to apixaban on 27 March. Injection of meropenem was started instead of ongoing injection of ceftriaxone. As urine output became almost nil on the 6th POD (Table 1), hemodialysis (HD) was initiated. At that time, the serum cyclosporine level (C2 level) was 1,576 ng/mL. Transplant biopsy was done on the 7th POD. On the same day, we changed cyclosporine to tacrolimus. Tacrolimus 3 mg 12 hourly was started (0.1 mg/kg/day). In the meantime, a donor-specific antibody (DSA) test was performed, and the result was negative. Subsequently, five sessions of HD were given on every alternate day (EAD) up to 14th POD. Renal biopsy report revealed ATN (light microscope, Figure 1), acute tubular injury (Banff category 6), and IHC marker: C4d non-immunoreactive.

On the 10th POD, the patient started improving, as indicated by increasing urine output and gradual decline of the serum creatinine level. The drain tube was removed on the 10th POD, and serum creatinine returned to baseline at the 20th POD (Table 1).

On the 27th POD, a Doppler study of the lower limbs again suggested post-thrombotic syndrome. Serum creatinine was 1.2 mg/dL, and blood pressure (BP) was controlled with four antihypertensive drugs. The patient was discharged on the 30th POD in stable condition, with baseline serum creatinine of 1.1 mg/dL.

## Discussion

ATN is a significant cause of DGF after renal transplantation and is associated with increased morbidity, prolonged hospital stays, and reduced long-term graft survival [8]. In our case, a 44-year-old male recipient of a living-related kidney transplantation developed ATN in the early postoperative period, complicated by deep vein thrombosis (DVT) and nephrotoxicity from cyclosporine, requiring multiple sessions of hemodialysis. Timely intervention with steroid pulse therapy, conversion to tacrolimus, and supportive care led to a gradual return of graft function. ATN is the leading cause of DGF, especially in cases involving ischemia-reperfusion injury and nephrotoxic medications [8,9].

Cyclosporine, a calcineurin inhibitor (CNI), though effective in reducing acute rejection, has well-documented nephrotoxic potential [10]. It induces vasoconstriction of the afferent arterioles and leads to direct tubular epithelial injury, predisposing patients to ATN [11]. This nephrotoxicity is more prominent in the early post-transplant period, especially when compounded by hemodynamic instability or thrombotic events, as seen in our patient [12]. Prompt conversion from cyclosporine to tacrolimus has been shown to mitigate further injury in selected cases [13]. In our case, the onset of DGF corresponded temporally with the development of a right lower limb DVT, possibly contributing to systemic inflammation and local hypoperfusion. The importance of thromboprophylaxis in high-risk post-transplant patients cannot be overemphasized, especially given the potential for thromboembolic complications to precipitate or exacerbate graft dysfunction [6].

Renal biopsy remains the gold standard for diagnosing the underlying pathology in DGF. In our case, biopsy confirmed ATN (Banff category 6) with no evidence of rejection (C4d-negative, DSA-negative), allowing us to avoid unnecessary escalation of anti-rejection therapy [4]. This aligns with studies emphasizing the role of biopsy in distinguishing ATN from acute rejection, which can have overlapping clinical features but require vastly different treatments [14]. The patient’s gradual improvement and recovery of renal function demonstrate the potential for reversibility in ATN with appropriate intervention. Nonetheless, the episode highlights the need for careful monitoring of CNI levels, early identification of vascular complications, and prompt adjustment of immunosuppressive regimens to optimize graft outcomes.

## Conclusions

This case underscores the vital importance of acting quickly when complications such as ATN arise after a kidney transplantation. In this case, early intervention, such as switching from cyclosporine to tacrolimus and starting dialysis, was key in reversing kidney dysfunction and helping the success of transplantation. It is a strong reminder that many transplant-related challenges can be resolved with the appropriate care at the appropriate time, giving patients the chance to recover and regain their graft function.
